# The interaction mechanisms between sarcopenia and gut microbiota based on the gut-muscle axis

**DOI:** 10.3389/fmed.2026.1892866

**Published:** 2026-10-27

**Authors:** Ya Ma, Yunpeng Sui, Xin Xia, Lixing Zhou, Jirong Yue, Birong Dong, Ning Ge, Xiaolei Liu

**Affiliations:** 1 National Clinical Research Center of Geriatrics, West China Hospital, Sichuan University, Chengdu, China; 2 Department of Geriatrics, West China Hospital, Sichuan University, Chengdu, China; 3 Plastic and Cosmetic Surgery Department, West China Tianfu Hospital, Sichuan University, Chengdu, China

**Keywords:** aging, gut microbiota, gut-muscle axis, mechanisms, sarcopenia

## Abstract

Advances in high-throughput sequencing and metagenomics have improved our understanding of the gut microbiota and its associations with disease development and treatment response. The relationship between the gut microbiota and sarcopenia has attracted increasing attention in recent years. In this narrative review, we searched PubMed, Web of Science, Embase, CNKI, Wanfang Data, and VIP through April 2,026 and synthesized 29 human studies included in the narrative analysis together with relevant preclinical evidence. We summarize reported alterations in microbial diversity, taxonomic composition, functional potential, and microbiota-related metabolites in community-based or age-related sarcopenia, disease-associated sarcopenia, and related muscle phenotypes. This narrative review aims to provide a mechanistic framework for understanding the gut-muscle axis and to identify potential directions for the prevention and management of sarcopenia.

## 1 Introduction

Sarcopenia represents a clinical syndrome typified by the gradual reduction in skeletal muscle mass, diminished muscular strength, and compromised physical performance (1). It increases the risk of adverse health outcomes and impairs quality of life, making it an important concern in aging populations (1, 2). Aging, nutrition, physical activity, and chronic diseases contribute to sarcopenia. The gut microbiota provides an additional perspective because microbial metabolism and host immune responses may influence muscle homeostasis (3, 4). Human studies have reported differences in gut microbial composition between individuals with and without sarcopenia, although the patterns vary across populations and methods and their contribution to muscle impairment remains uncertain. This narrative review examines proposed gut–muscle mechanisms, compares microbial and metabolite findings in human studies, and evaluates microbiota-targeted interventions. We distinguish human observational and intervention evidence from animal and *in vitro* findings to clarify the limits of causal interpretation and clinical translation.

### 1.1 Literature identification and evidence selection

We conducted a narrative review rather than a systematic review or meta-analysis. PubMed, Web of Science, Embase, CNKI, Wanfang Data, and VIP were searched through April 2026 using combinations of terms related to “sarcopenia,” “muscle mass,” “muscle strength,” “gut microbiota,” “gut microbiome,” “intestinal flora,” “gut–muscle axis,” and relevant microbial metabolites. Reference lists of relevant original studies and reviews were also examined.

The search aimed to identify clinically and mechanistically informative literature rather than an exhaustive set of eligible studies. Human reports were considered relevant when they examined gut microbial composition, microbial metabolites, or microbiota-related pathways in relation to sarcopenia or related muscle phenotypes. Reports in Table 1 are organized by study population and the diagnosis or phenotype described: (i) sarcopenia in general older populations, including reports with possible sarcopenia or comparisons between muscle-mass strata; (ii) sarcopenia in disease-specific populations, including chronic kidney disease, liver cirrhosis, heart failure, and maintenance hemodialysis; and (iii) explicitly defined isolated muscle phenotypes, such as low muscle mass, low skeletal muscle index, muscle wasting, or low handgrip strength. The first category does not establish that aging was the sole cause of sarcopenia, and its heterogeneous operational definitions should not be treated as equivalent. Disease-specific and isolated-phenotype findings provide contextual evidence and are identified as such in the synthesis. Differences in diagnostic definitions, comorbidities, medication use, and nutrition limit comparability across reports (1, 5–7).

**Table 1:** Characteristics, diagnostic criteria, population groups, and principal findings of 29 human research reports on gut microbiota in sarcopenia and related muscle phenotypes.

Report (Ref.)	Population	Age (years)	Study design	Diagnostic criterion/phenotype definition	Study groups	Microbiota assessment	Principal findings
Category I. Sarcopenia in general older populations (20 reports).							
Zhou et al. (37)	Sarcopenic individuals	83.5–92.0	Observational	AWGS 2019	① Sarcopenic: *n* = 30; ② Not Sarcopenic: *n* = 30	Feces, 16S rRNA	Patients with sarcopenia: - ↑: Acetatifactor, Limosilactobacillus - ↓: Firmicutes, Subdoligranulum, Clostridia_UCG_014_unclassified, Blautia
Wang et al. (38)	Elderly women with sarcopenia	68.7 ± 3.4	Observational	EWGSOP2 (2018) + AWGS 2019 (stepwise screening)	① sarcopenic: *n* = 50; ② not sarcopenic: *n* = 50	Feces, Metagenomics	Elderly women with sarcopenia: - ↑: Eggerthella lenta, Collinsella aerofaciens, Subdoligranulum variabile - ↓: Bacteroides fluxus, Barnesiella intestinihominis, Bacteroides coprocola, Bacteroidales bacterium (ph8), Bacteroides massiliensis, Mitsuokella multacida, Bacteroides coprophilus, gammaretrovirus
Ticinesi et al. (39)	Sarcopenic individuals	75.5–86	Observational	PF&S: SPPB 3–9 + low SMI/height^2^ (<8.87 kg/m^2^ men; <6.42 kg/m^2^ women; EWGSOP1 cut-offs)	① Sarcopenic: *n* = 5; ② Not Sarcopenic: *n* = 12	Feces, Shallow-shotgun metagenomics	Patients with sarcopenia: - ↓: Faecalibacterium prausnitzii, Roseburia inulinivorans, Alistipes shahii
Picca et al. (40)	Older adults with physical frailty and sarcopenia	75.5 ± 3.9	Observational	SPRINTT PF&S: SPPB 3–9 + low appendicular lean mass (FNIH cut-offs) + ability to complete 400 m walk	① PF&S subjects: *n* = 18; ② Non-PF&S controls: *n* = 17	Feces, 16S rRNA	Older adults with physical frailty and sarcopenia: - ↑: Oscillospira, Ruminococcus, Peptostreptococcaceae, Bifidobacteriaceae, Dialister, Pyramidobacter, Eggerthella - ↓: Barnesiellaceae, Christensenellaceae, Eubacterium, Slackia
Kang et al. (41)	Patients with sarcopenia and possible sarcopenia	NR	Observational	AWGS 2019	① Sarcopenia: *n* = 11; ② Possible sarcopenia: *n* = 16; ③ Healthy controls: *n* = 60	Feces, 16S rRNA	Patients with sarcopenia and possible sarcopenia: - ↑: Lactobacillus - ↓: Lachnospira, Fusicantenibacter, Roseburia, Eubacterium, Lachnoclostridium
He et al. (42)	Elderly people with sarcopenia	62–88	Observational	AWGS 2019	① Sarcopenic: *n* = 32; ② Not Sarcopenic: *n* = 31	Feces, Shotgun metagenomics	Elderly people with sarcopenia: - ↑: Clostridium sp. CAG 242, Clostridium sp. CAG 58, Phascolarctobacterium succinatutens - ↓: Bifidobacterium longum, Bifidobacterium pseudocatenulatum, Bifidobacterium adolescentis, Faecalibacterium prausnitzii, Fusicatenibacter saccharivorans, Phascolarctobacterium faecium
Guan et al. (43)	Elderly people with sarcopenia	72.00 ± 10.68	Observational	AWGS 2019	① Sarcopenic: *n* = 7; ② Not Sarcopenic: *n* = 8	Feces, 16S rRNA	Elderly people with sarcopenia: - ↓: Tannerellaceae, unclassified_k__norank_d__Bacteria, Eubacteriaceae
Liu et al. (20)	Elderly people with sarcopenia	69.10 ± 8.00	Observational	AWGS 2014	① Sarcopenic: *n* = 141; ② Not Sarcopenic: *n* = 142	Feces, Metagenomics	Elderly people with sarcopenia: - ↑: Bacteroides vulgatus, Bifidobacterium, Roseburia, Faecalibacterium prausnitzii - ↓: Prevotella copri, Eubacterium rectale
Lou et al. (44)	Patients with sarcopenia	72.5 ± 4.7	Observational	RSMI <7.26 kg/m^2^ (men) or <5.45 kg/m^2^ (women); low-muscle-mass thresholds as tabulated	① Sarcopenic: *n* = 108; ② Not Sarcopenic: *n* = 98	Feces, 16S rRNA	Patients with sarcopenia: - ↑: Escherichia-Shigella - ↓: Lactobacillus, Lachnospira, Eubacterium rectale, Faecalibacterium prausnitzii
Shan et al. (45)	Elderly Uygur patients with sarcopenia	76.52 ± 7.40	Observational	AWGS criteria	① Sarcopenic: *n* = 40; ② Not Sarcopenic: *n* = 40	Feces, 16S rRNA	Elderly Uygur patients with sarcopenia: - ↑: Lachnoclostridium, Photobacterium, Anaerobic Bacillus, Hydrogenophilus, Eubacterium, Bacillus, Vibrionaceae - ↓: Prevotella 9, Firmicutes FCS020 group, Streptobacillus, Aggregatibacter, Corynebacterium, Clostridium difficile, Haloanaerobium, Corynebacteriaceae
Wang et al. (46)	Participants with sarcopenia	72.2 ± 8.5	Observational	AWGS 2019	① Sarcopenic: *n* = 141; ② Not Sarcopenic: *n* = 1276	Feces, Shotgun metagenomics	Patients with sarcopenia: - ↑: Clostridium, Lawsonibacter, Desulfovibrio piger, Clostridium symbiosum, Hungatella effluvii, Bacteroides fluxus, Absiella innocuum, Coprobacter secundus, Clostridium citroniae
Yan et al. (47)	Elderly women with sarcopenia	75.26 ± 7.14	Observational	AWGS 2019	① Sarcopenic: *n* = 53; ② Not Sarcopenic: *n* = 223	Feces, 16S rRNA	Elderly women with sarcopenia: - ↑: Shigella, Bacteroides - ↓: Firmicutes/Bacteroidetes, Agathobacter, Dorea
Zhang et al. (48)	Uyghur individuals with sarcopenia	55.39 ± 7.59	Observational	AWGS 2019	① Sarcopenic: *n* = 31; ② Not Sarcopenic: *n* = 31	Feces, 16S rRNA	Uyghur individuals with sarcopenia: - ↑: Flavobacteriales, Flavobacteriaceae, Catenibacterium, Romboutsia, Erysipelotrichaceae_UCG-003, GCA-900066575, Lachnospiraceae_FCS020_group, un_f_Flavobacteriaceae - ↓: Alloprevotella, un_f_Prevotellaceae, Anaerovibrio, Prevotellaceae_NK3B31_group, Mitsuokella, Prevotella, Allisonella
Zhang et al. (49)	Patients with sarcopenia	71.21 ± 6.85	Observational	AWGS 2019	① Sarcopenic: *n* = 14; ② Not Sarcopenic: *n* = 21	Feces, 16S rRNA	Patients with sarcopenia: - ↑: Escherichia-Shigella, Blautia, Klebsiella - ↓: Bacteroides, Faecalibacterium, Fusobacterium, Prevotella 9
Lee et al. (50)	Patients with sarcopenia	66.5 ± 4.6	Observational	AWGS 2019	① Sarcopenic: *n* = 27; ② Not Sarcopenic: *n* = 33	Feces, 16S rRNA	Patients with sarcopenia: - ↑: Parabacteroides sp. - ↓: Prevotella, Prevotella copri, Anaerotruncus, Butyricimonas, Phascolarctobacterium, Ruminococcaceae_unclassified, Parabacteroides, unclassified species
Asaoka et al. (51)	Patients with sarcopenia	79.5 ± 6.9	Observational	AWGS 2019	① Sarcopenic: *n* = 50; ② Not Sarcopenic: *n* = 306	Feces, 16S rRNA	Patients with sarcopenia: - ↓: Eubacterium I, Fusicatenibacter, Holdemanella, Unclassified Lachnospira, Enterococcus H, Bariatricus
Wang et al. (52)	Patients with sarcopenia	72.56 ± 3.93	Observational	AWGS 2019	① Sarcopenic: *n* = 18; ② Sarcopenic Obesity: *n* = 20; ③ Not Sarcopenic: *n* = 22	Feces, 16S rRNA	Patients with sarcopenia: - ↑: Christensenellaceae_R-7_group, Alistipes, Ruminococcus, Odoribacter, Prevotellaceae_UCG-001, Hungatella, Family_XIII_AD3011_group, Anaerotruncus, Ruminiclostridium, Oxalobacter Patients with sarcopenic obesity: - ↑: Enterobacteriaceae, Allisonella, Peptoclostridium
Li et al. (53)	Older patients reported to have sarcopenia	85 ± 4	Observational	AWGS 2014	Elderly males: ① Very low SMI group: *n* = 43; ② Low SMI group: *n* = 38	Feces, PCR-based targeted bacterial quantification	Elderly males in the very low SMI group: - ↓: Faecalibacterium prausnitzii, Clostridium cluster I Elderly females in the very low SMI group: - ↑: Clostridium cluster I - ↓: Enterococcus
					Elderly females: ① Very low SMI group: *n* = 11; ② Low SMI group: *n* = 11		
Lou et al. (54)	Elderly patients with sarcopenia	72.5 ± 4.7	Observational	AWGS 2019	① sarcopenia group: *n* = 108; ② Normal group: *n* = 98	Feces, 16S rRNA	Elderly sarcopenia group: - ↑: Escherichia coli - ↓: Lachnoclostridium, Fusobacterium, Rothia, Lactobacillus, Eubacterium rectale, Cyanobacteria
Karolina & Yang (55)	Elderly patients with sarcopenia	NR	Observational	AWGS criteria (version not specified)	① sarcopenia group: *n* = 48; ② Normal group: *n* = 11	Feces, 16S rRNA	Elderly patients with sarcopenia group: - ↓: Enterobacteriaceae bacteria
Category II. Disease-associated sarcopenia (5 reports).							
Margiotta et al. (56)	Elderly CKD patients with sarcopenia	83.1 ± 5.7	Observational	EWGSOP2 (2018)	① Sarcopenic: *n* = 18; ② Not Sarcopenic: *n* = 45	Feces, 16S rRNA	Older CKD patients with sarcopenia: - ↑: Micrococcaceae, Verrucomicrobiaceae, Megasphaera, Rothia, Veillonella, Akkermansia, Coprobacillus - ↓: Gemellaceae, Veillonellaceae, Acidaminococcus, Gemella
Peng et al. (57)	Heart failure patients with sarcopenia	75.1 ± 8.18	Observational	AWGS 2019	① HF patients with sarcopenia: *n* = 29; ② HF patients without sarcopenia: *n* = 33; ③ Control individuals: *n* = 15	Feces, 16S rRNA	Heart failure patients with sarcopenia: - ↑: Lactobacillus, Bifidobacterium, Enterococcus, Proteobacteria, Actinobacteria - ↓: Shigella, Bacteroides, Streptococcus, Gemmiger, Akkermansia, Faecalibacterium, Blautia, Prevotella
Ponziani et al. (58)	Cirrhotic patients with sarcopenia	70 (63–74)	Observational	FNIH-based sarcopenia criteria (muscle mass + strength)	① Cirrhotic patients with sarcopenia: *n* = 19; ② Cirrhotic patients without sarcopenia: *n* = 31; ③ Controls with sarcopenia: *n* = 14; ④ Controls without sarcopenia: *n* = 36	Feces, 16S rRNA	Cirrhotic patients with sarcopenia: - ↑: Eggerthella, Klebsiella - ↓: Methanobrevibacter, Prevotella, Akkermansia
Zhou et al. (59)	MHD patients with sarcopenia	49.9 ± 12.6	Observational	AWGS 2019	① Sarcopenic: *n* = 30; ② Not Sarcopenic: *n* = 30	Feces, 16S rRNA	MHD patients with sarcopenia: - ↑: Tyzzerella_4 - ↓: Megamonas, Coprococcus_2, uncultured_bacterium_f_Muribaculaceae
Aliwa et al. (60)	Cirrhotic patients with sarcopenia	64 (61–68)	Observational	EWGSOP 2010	① Cirrhotic patients with sarcopenia: *n* = 78; ② Cirrhotic patients without sarcopenia: *n* = 38; ③ Controls with sarcopenia: *n* = 39; ④ Controls without sarcopenia: *n* = 20	Feces, 16S rRNA	Patients with sarcopenia: - ↑: Bacteroides fragilis, Blautia marseille, Sutterella spp., Veillonella parvula - ↓: Bacteroides ovatus
Category III. Isolated muscle phenotypes (4 reports).							
Han et al. (61)	Participants with low muscle mass	72.3 ± 5.4	Observational	Low muscle mass phenotype: IWGS criterion	① Low muscle mass group: *n* = 36; ② Normal muscle mass group: *n* = 52	Feces, 16S rRNA	Participants with low muscle mass: - ↑: Flavonifractor - ↓: Marvinbryantia, Ruminococcaceae UCG-10, Akkermansia
Ren et al. (62)	Liver cirrhosis with muscle wasting	45.5 ± 12.74	Observational	Muscle-wasting phenotype: L3 SMI <52.4 cm^2^/m^2^ (men) or <38.5 cm^2^/m^2^ (women)	① Healthy controls: *n* = 30; ② Liver cirrhosis with muscle wasting: *n* = 30; ③ Liver cirrhosis without muscle wasting: *n* = 30	Feces, Metagenomics	Patients with liver cirrhosis and muscle wasting: - ↑: B. faecis, E. infirmum, Weissella, B. caccae, B. coprocola, E. coli, P. stomatis - ↓: Bacteroides uniformis, Clostridium clostridioforme, C. asparagiforme, Ruminococcus flavefaciens, Lachnospiraceae bacterium 5 1 63FAA, Granulicella unclassified, Anaerostipes hadrus, Eggerthella unclassified, Lachnospiraceae bacterium 7 1 58FAA, Bifidobacterium catenulatum, Erysipelotrichaceae bacterium 2 2 44A
Yamamoto et al. (63)	Patients with chronic liver disease and low skeletal muscle index	68 (62.0–73.3)	Observational	Low-SMI phenotype: JSH guideline (1st ed.): L3 SMI <42 cm^2^/m^2^ (men) or <38 cm^2^/m^2^ (women)	① L-SMI: *n* = 25	Feces, 16S rRNA	L-SMI: - ↑: Bacteroidetes, Bacteroides - ↓: Firmicutes, Prevotella, Coprobacillus, Catenibacterium, Clostridium, Firmicutes to Bacteroidetes ratio
					② N-SMI: *n* = 44		
Guo et al. (64)	Patients with low handgrip strength	81.57 ± 3.15	Observational	Low-HGS phenotype: AWGS 2019 HGS cut-offs (<28 kg men; <18 kg women)	① Low HGS: *n* = 8	Feces, 16S rRNA	Patients with low handgrip strength: - ↑: Parabacteroides, Intestinibacter, Bacteroidaceae, Bacteroides, Selenomonadales, Negativicutes, Porphyromonadaceae, Enterococcus, Enterococcaceae - ↓: Sporobacter, Terrisporobacter
					② Normal HGS: *n* = 7		

Studies are grouped according to the three-category framework used in this review: Category I, sarcopenia in general older populations; Category II, disease-associated sarcopenia; and Category III, sarcopenia-related phenotypes. Reference numbers correspond to the numbered reference list.

AWGS, Asian Working Group for Sarcopenia; EWGSOP, European Working Group on Sarcopenia in Older People; FNIH, Foundation for the National Institutes of Health; PF&S, physical frailty and sarcopenia; IWGS, International Working Group on Sarcopenia; JSH, Japan Society of Hepatology; SMI, skeletal muscle index; RSMI, relative skeletal muscle mass index; HGS, handgrip strength; SPPB, Short Physical Performance Battery; MHD, maintenance hemodialysis; CKD, chronic kidney disease; HF, heart failure; NR, not reported.

For Table 1, two authors extracted information on population, age, study design, diagnostic criteria or phenotype definition, sample size, microbiota assessment, and principal findings; disagreements were resolved through discussion with another author. The table contains 29 representative research reports, not a confirmed count of independent cohorts. Potential participant overlap across reports may affect the apparent consistency of findings. Additional human mechanistic and intervention studies are discussed outside the table. No review protocol was registered, and no formal systematic risk-of-bias assessment was performed. The synthesis is therefore qualitative and hypothesis-generating.

## 2 Mechanistic links between gut microbiota and sarcopenia

Functional communication between the gut microbiota and skeletal muscle is mediated largely by microbial metabolites, host-microbial co-metabolites, and inflammatory mediators. These factors can influence substrate utilization, protein turnover, mitochondrial metabolism, and regenerative responses in muscle, although the strength and completeness of the evidence vary across pathways. Human studies have primarily identified associations among microbial functions, circulating metabolites, and muscle phenotypes, whereas animal and cell experiments have examined the effects of individual mediators and their downstream signaling pathways. Mechanistic interpretation of the gut-muscle axis therefore requires consideration of mediator origin, host exposure, molecular responses in muscle, and functional outcomes. Changes in common muscle signaling pathways alone are insufficient to establish microbial involvement. Figure 1 summarizes the candidate mechanisms discussed in Sections 2.1–2.6 and highlights unresolved links in the evidence.

**Figure 1:**
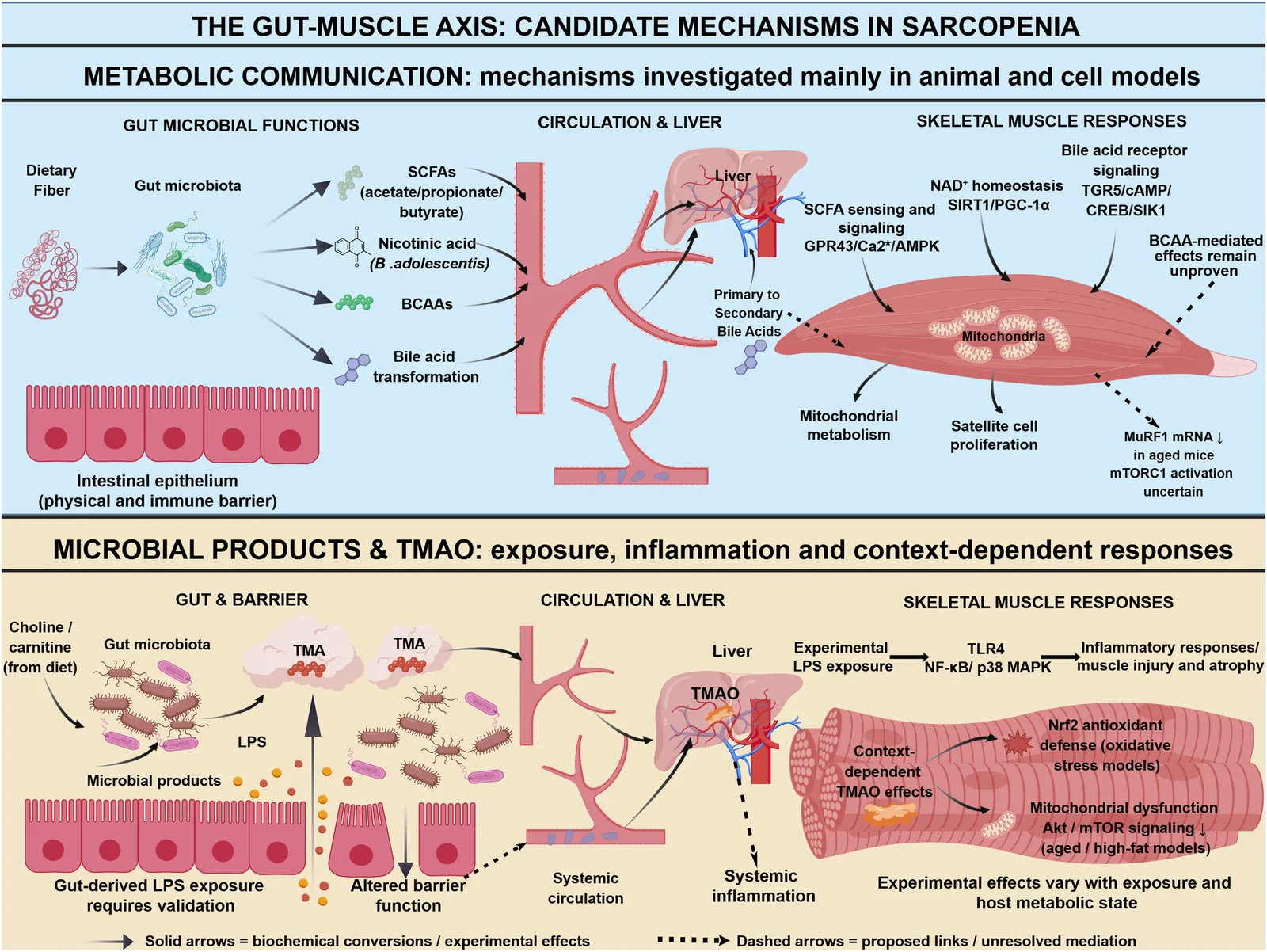
Candidate mechanisms linking the gut microbiota to skeletal muscle in sarcopenia. The upper panel summarizes pathways involving SCFAs, nicotinic acid, BCAAs, and bile acid signaling. The lower panel depicts LPS-related inflammatory responses and context-dependent effects of TMAO. Solid arrows indicate biochemical conversions or experimental effects; dashed arrows indicate proposed links or unresolved mediation. Mechanistic evidence derives mainly from animal and cell studies and does not establish causality in human sarcopenia. See Sections 2.1–2.6 for supporting studies and pathway definitions. This figure was created using the Generic Diagramming Platform (GDP) (36).

### 2.1 Microbial fermentation and short-chain fatty acid-mediated regulation of skeletal muscle

Short-chain fatty acids (SCFAs), principally acetate, propionate, and butyrate, are produced through microbial fermentation of dietary carbohydrates. Their production depends on substrate availability, microbial metabolic capacity, and cross-feeding between community members (8). For example, acetate released by bifidobacteria can be utilized by *Faecalibacterium prausnitzii*, while strain-specific patterns of substrate utilization and competition influence the final butyrate yield (9, 10). These interactions make the collective fermentative activity of the gut microbiota an important link between diet and host metabolism. Following intestinal absorption and host metabolism, a proportion of SCFAs reaches the systemic circulation and becomes available to peripheral tissues (11). A human stable-isotope study estimated systemic availability at approximately 36% for acetate, 9% for propionate, and 2% for butyrate under the colonic administration conditions examined, indicating substantial differences in systemic delivery (11). Microbial fermentation potential, fecal concentrations, and circulating concentrations thus capture distinct stages of SCFA production and disposition. Studies integrating microbial functional profiles with circulating metabolite measurements may consequently provide a clearer understanding of SCFA-muscle relationships.

Human multi-omics studies have provided population-level evidence for these relationships. In a study of 482 healthy, community-dwelling postmenopausal Chinese women, a selected microbial functional measure related to butyrate synthesis was positively associated with serum butyrate and skeletal muscle index. *F. prausnitzii* and *Butyricimonas virosa* were also associated with related measures (12). One-sample Mendelian randomization supported a potential causal relationship between this functional measure and appendicular lean mass, but not skeletal muscle index. The two-sample analysis did not meet the conventional threshold for statistical significance (*P* = 0.06) (12). These findings connect microbial metabolic function and circulating butyrate with muscle mass, providing a basis for further mechanistic investigation.

Animal experiments have further identified SCFAs as candidate mediators of microbial effects on skeletal muscle. Lahiri et al. reported reduced muscle mass and strength in germ-free mice, with partial reversal of muscle abnormalities after microbial colonization (13). SCFA supplementation also improved muscle strength and reduced Atrogin-1 expression in the tibialis anterior. In dexamethasone-treated mouse C2C12 myotubes, SCFAs attenuated the increase in Atrogin-1 expression, supporting a role in the regulation of atrophy-associated responses (13). However, muscle RING finger protein 1 (MuRF1) expression increased in the same animal study, and some increases in mitochondrial gene expression observed *in vitro* were not reproduced in mouse muscle (13). These discrepancies suggest that the effects of SCFAs differ across biological processes within skeletal muscle.

Receptor-based studies offer a molecular explanation for some of these effects. In rat L6 myotubes, acetate increased intracellular calcium concentrations, accompanied by changes in calcineurin activity and the nuclear localization of myocyte enhancer factor 2A (MEF2A), peroxisome proliferator-activated receptor gamma coactivator 1-alpha (PGC-1α), and nuclear factor of activated T cells 1 (NFATc1) (14). G protein-coupled receptor 43 (GPR43) silencing attenuated a subset of these responses, whereas phospholipase C inhibition reduced calcium signaling and AMP-activated protein kinase (AMPK) phosphorylation (14). These interventions implicate GPR43 and associated calcium signaling in acetate-induced muscle cell responses, linking SCFA sensing to transcriptional regulation of muscle metabolism. An earlier study in L6 myotubes also found that acetate treatment increased AMPK phosphorylation, GLUT4 expression, and glucose and fatty acid uptake (15). Beyond receptor-mediated signaling, SCFAs can directly contribute to substrate metabolism in muscle cells. Primary myotubes derived from healthy donors took up and oxidized labeled acetate and butyrate. Butyrate increased leucine accumulation and incorporation into protein, while also altering oleate uptake and oxidation and the metabolic partitioning of leucine-derived carbon. Basal and insulin-stimulated glucose metabolism remained largely unchanged (16). These findings in human-derived cells indicate that SCFAs may directly influence muscle substrate utilization and highlight distinct responses of fatty acid, amino acid, and glucose metabolism to the same stimulus (16). Complementary evidence from high-fat-diet-fed mice showed that dietary butyrate supplementation increased skeletal muscle mitochondrial function and biogenesis and elevated PGC-1α expression, although these findings were obtained in a metabolic disease model rather than age-related sarcopenia (17).

Studies in naturally aging animals suggest that prolonged butyrate exposure may help preserve muscle structure and oxidative metabolism. Walsh et al. fed female mice a diet containing 5% sodium butyrate from 16 months of age. At 26 months, treated mice exhibited less muscle loss and intramuscular fat accumulation, together with increases in selected indices of oxidative metabolism and in mitochondrial transcription factor A (TFAM) and porin levels (18). PGC-1α levels were not significantly altered, and the age-related decline in grip strength was not improved (18). Thus, the structural and metabolic benefits observed in this model did not translate into a corresponding recovery of muscle strength.

Collectively, the available evidence indicates that SCFAs can influence muscle protein turnover and energy metabolism through receptor signaling and direct participation in substrate metabolism (14, 16, 18). Their effects depend on metabolite identity, exposure conditions, and host physiological state (18). Human evidence largely concerns associations among microbial functions, circulating metabolites, and muscle mass, whereas detailed mechanistic support comes predominantly from animal and cell studies (13, 17, 18). Future human intervention studies should jointly assess microbial functions, circulating SCFA concentrations, muscle metabolism, and strength to determine the contribution of these experimental mechanisms to sarcopenia.

### 2.2 Microbial nicotinic acid metabolism and muscle NAD⁺ homeostasis

Microbially derived nicotinic acid provides a specific link between the gut microbiota and oxidized nicotinamide adenine dinucleotide (NAD⁺) metabolism in skeletal muscle. A study integrating human metagenomics, metabolomics, and experimental validation found that individuals with sarcopenia had a lower abundance of *Bifidobacterium adolescentis* and that this lower abundance was associated with poorer measures of muscle health. Nicotinic acid produced by this bacterium was subsequently identified as a candidate effector, connecting species-level microbial variation with muscle metabolism (19).

In aged mice, supplementation with either *B. adolescentis* or nicotinic acid improved muscle mass and function and was accompanied by restoration of muscle NAD⁺ levels (19). Downstream changes included suppression of atrophy-related signaling involving forkhead box O3 (FoxO3), Atrogin-1, and MuRF1, together with enhancement of sirtuin 1 (SIRT1)/PGC-1α-associated signaling. These changes were accompanied by improvements in mitochondrial abundance and function and increased adenosine triphosphate (ATP) content (19). These findings link the effects of this microbial metabolite to both protein turnover and oxidative metabolism in muscle.

Cell experiments provided additional evidence for direct effects of nicotinic acid. Dexamethasone-treated C2C12 cells were used to examine changes in NAD⁺ levels and metabolic responses, while experiments in muscle satellite cells demonstrated improved proliferation. These findings suggest that nicotinic acid may support muscle regenerative capacity in addition to contributing to myofiber homeostasis, although enhanced satellite cell proliferation alone does not establish improved regeneration following muscle injury (19).

By combining human microbial associations with bacterial and metabolite interventions in animals and functional experiments in cells, this work provides a relatively coherent mechanistic framework for microbial regulation of muscle NAD⁺ homeostasis. Nevertheless, evidence in humans remains observational, whereas intervention effects have been demonstrated in preclinical models. Human studies are needed to establish whether supplementation with the target bacterium increases nicotinic acid availability and skeletal muscle NAD⁺ levels, and whether these changes translate into improved strength and physical function.

### 2.3 Microbial branched-chain amino acid metabolism and muscle protein turnover

Microbiota-associated branched-chain amino acid (BCAA) metabolism represents a potential connection between the gut microbiota and muscle protein homeostasis. Studies have examined differences in microbial functions and metabolite profiles and subsequently used species-specific interventions to investigate changes in host amino acid availability and muscle phenotypes. This approach provides a basis for understanding how microbial activity may influence the supply of nutritional substrates to muscle (20).

In a multiethnic cross-sectional study of 283 participants, Liu et al. found that individuals with sarcopenia had a lower abundance of *Prevotella copri* and lower fecal levels of selected BCAAs and their derivatives. BCAA metabolism-related functions were enriched in participants without sarcopenia (20). In a subsequent eight-week intervention in 20-month-old mice, oral gavage with live *P. copri* increased circulating BCAA levels and gastrocnemius and rectus femoris weights and improved performance in grip strength, hanging, and rotarod tests (20). At the molecular level, muscle MuRF1 mRNA expression decreased, whereas Atrogin-1 expression showed a downward trend that did not reach statistical significance (20). The expression of selected genes associated with protein synthesis was not significantly altered. This study connects differences in microbial abundance and metabolite profiles observed in humans with beneficial muscle phenotypes following bacterial supplementation in aged mice. However, the downstream molecular evidence primarily concerns markers of protein degradation. The results therefore suggest increased circulating BCAA availability accompanied by changes in muscle protein degradation markers. They do not directly establish enhanced protein synthesis through mechanistic target of rapamycin complex 1 (mTORC1). Whether higher circulating BCAA levels translate into greater amino acid uptake and incorporation into skeletal muscle protein remains a critical unresolved link between the observed metabolic changes and muscle benefits (20).

The effects of BCAA metabolism also depend on the host metabolic context. In a human study, Pedersen et al. associated microbial BCAA biosynthetic potential with insulin resistance and identified *P. copri* and *Bacteroides vulgatus* as major contributors to this biosynthetic potential. In the accompanying mouse experiments, *P. copri* increased circulating BCAAs but also worsened glucose intolerance and insulin resistance. These findings differ from those reported in aged muscle models, indicating that increases in bacterial abundance or circulating BCAAs must be interpreted in relation to diet, strain identity, and host metabolic status (20, 21).

Current evidence supports a possible role for *P. copri*-associated BCAA metabolism in gut-muscle communication. However, human findings remain predominantly observational, and evidence for muscle protection and downstream molecular responses comes mainly from animal experiments. Future strain intervention studies should jointly characterize amino acid sources, their utilization by skeletal muscle, and protein turnover dynamics. Such measurements are needed to distinguish BCAAs that accompany phenotypic changes from those that functionally mediate muscle benefits.

### 2.4 Microbial bile acid transformation and receptor signaling in the intestine and skeletal muscle

By transforming bile acids, the gut microbiota modifies the host bile acid pool and associated receptor signaling, with potential consequences for skeletal muscle metabolism. Two experimentally investigated routes are: regulation of gut-derived endocrine signals through intestinal farnesoid X receptor (FXR) and modulation of myogenic responses through the G protein-coupled bile acid receptor 1, commonly known as TGR5, in skeletal muscle (22, 23). These routes provide complementary perspectives on bile acid–dependent communication between the gut and muscle.

Using an antibiotic-induced microbiota depletion model in mice, Qiu et al. found that disruption of microbial bile acid transformation caused tauro-β-muricholic acid accumulation, suppressed ileal FXR–fibroblast growth factor 15 (FGF15) signaling, and reduced circulating FGF15 (22). In skeletal muscle, phosphorylation of extracellular signal-regulated kinases 1 and 2 (ERK1/2), p90 ribosomal S6 kinase (p90RSK), and ribosomal protein S6 (RPS6) decreased, while puromycin incorporation assays demonstrated reduced protein synthesis. Supplementation with fibroblast growth factor 19 (FGF19), the human ortholog of mouse FGF15, partially restored these signaling responses and protein synthesis, increased muscle mass, and improved grip strength (22). These findings connect microbial perturbation, altered bile acid composition, changes in a gut-derived endocrine mediator, and muscle anabolism. However, mechanistic support was obtained through supplementation with a downstream mediator, and muscle-specific receptor knockout models were not used to exclude indirect effects of FGF19 through other organs. Moreover, the bile acid pool differs between mice and humans, so the relevance of the observed bile acid changes requires human validation (22).

Studies of muscle TGR5 provide further insight into downstream bile acid signaling. Sasaki et al. showed that taurolithocholic acid enhanced cyclic adenosine monophosphate (cAMP) response element (CRE)–dependent transcription and increased the expression of the salt-inducible kinase 1 gene (*Sik1*) and selected myogenic differentiation markers in TGR5-overexpressing C2C12 cells. Silencing *Sik1* attenuated some differentiation responses (23). *In vivo*, TGR5-deficient mice exhibited reduced muscle mass and strength, whereas muscle-specific TGR5 overexpression induced hypertrophy and increased muscle strength in both young and aged mice. These results support the involvement of TGR5-dependent signaling through cAMP, cAMP response element-binding protein (CREB), and salt-inducible kinase 1 (SIK1) in muscle differentiation and growth (23).

Together, these studies support bile acid-dependent regulation of skeletal muscle at different levels. Work on the FXR-FGF15/19 pathway links upstream microbial perturbation to changes in a circulating endocrine mediator and muscle anabolic responses, whereas TGR5 experiments primarily establish receptor function in muscle. The latter findings do not, by themselves, establish that microbiota-driven changes in bile acids activate this pathway *in vivo*. Bile acid effects should therefore be interpreted according to the specific molecule, the tissue in which its receptor is expressed, and the experimental setting. Neither increased secondary bile acid levels nor activation of an individual receptor can be assumed to confer muscle protection across contexts. As mechanistic evidence remains largely preclinical, human studies should connect microbial bile acid-transforming capacity, circulating bile acid profiles, and muscle phenotypes to clarify the contribution of this pathway to sarcopenia.

### 2.5 Exposure to microbial products and inflammation-mediated muscle injury

Microbial components and the host inflammatory responses they elicit constitute another route through which the gut microbiota may influence skeletal muscle. This pathway depends primarily on intestinal barrier integrity, systemic exposure to microbial products, and muscle responses to inflammatory stimuli. Evidence is comparatively extensive for lipopolysaccharide (LPS), while interventions targeting individual bacterial strains provide additional experimental support for links between microbial changes and muscle inflammation.

In a mouse model of acute leukemia, Bindels et al. observed that reduced abundance of certain lactobacilli was associated with increased inflammation and expression of muscle atrophy markers. Supplementation with a mixture of *Lactobacillus reuteri 100–23* and *Lactobacillus gasseri 311476* reduced circulating interleukin-6 (IL-6) and monocyte chemoattractant protein-1 (MCP-1) levels and decreased muscle mRNA expression of Atrogin-1, MuRF1, and selected markers of the autophagy-lysosome pathway (24). This bacterial intervention links changes in the gut microbiota to the host inflammatory environment and muscle catabolic responses. However, these findings concern cancer-associated muscle wasting, and their applicability to primary age-related sarcopenia remains to be established (24).

LPS exposure experiments have further characterized the molecular pathways through which microbial products affect muscle. In C2C12 myotubes, LPS activated Toll-like receptor 4 (TLR4)–mediated inflammatory responses, increased nuclear factor kappa B (NF-κB) activity and the expression of IL-6 and tumor necrosis factor-alpha (TNF-α), and increased protein degradation markers alongside myotube atrophy. Endotoxin exposure in mice similarly induced muscle inflammation, atrophy, and reduced grip strength. Pretreatment with the TLR4 inhibitor TAK-242 attenuated these abnormalities, providing pharmacological evidence for TLR4 involvement in LPS-induced muscle injury (25).

LPS can also act directly on muscle cells. In C2C12 myotubes, Doyle et al. demonstrated TLR4- and p38 MAPK-dependent catabolic responses and myotube atrophy despite concurrent Akt activation (26). Separately, Tarabees et al. found that LPS transiently inhibited protein synthesis in C2C12 myotubes, with changes involving Akt/mTOR and MAPK signaling (27). These findings concern different readouts and experimental conditions and should not be combined into a single obligatory signaling sequence. Together, they support direct muscle-cell responses to LPS in addition to indirect effects mediated by systemic inflammation.

Human intervention studies provide limited clinical evidence. A randomized controlled trial of a multicomponent supplement containing β-hydroxy-β-methylbutyrate (HMB), carnosine, magnesium, butyrate, and lactoferrin reported improvements in muscle mass and physical function alongside reductions in C-reactive protein (CRP), TNF-α, and zonulin levels (28). These concurrent changes suggest that muscle outcomes, inflammatory markers, and intestinal barrier–related markers can respond to the same intervention. However, the formulation contained several active ingredients with potential direct effects on muscle, preventing attribution of the benefits specifically to the microbiota or intestinal barrier pathways. The observed marker changes also do not establish that reduced inflammation or improved barrier function mediated the muscle benefits (28).

Bacterial supplementation, microbial product exposure, and receptor inhibition studies collectively support inflammatory signaling as a component of gut-muscle communication. Although the downstream effects of LPS-TLR4 signaling are well supported in cells and animals, these experiments do not, by themselves, establish the intestinal origin or physiological extent of LPS exposure in sarcopenia. Studies are still needed to connect microbiota disruption, altered intestinal barrier function, systemic exposure to microbial products, and muscle outcomes within a unified experimental framework. In addition, Atrogin-1, MuRF1, and autophagy-related markers provide information about catabolic pathways but do not, by themselves, measure protein degradation rates or autophagic flux. Combining these molecular readouts with functional measurements of protein turnover, muscle mass, and strength would allow more precise assessment of the contribution of inflammation to sarcopenia.

### 2.6 Microbial-host TMA/TMAO co-metabolism and muscle function

Trimethylamine N-oxide (TMAO) is a microbial-host co-metabolite. Gut microorganisms convert dietary substrates, including choline and carnitine, into trimethylamine (TMA), which is subsequently oxidized to TMAO in the liver. Understanding the relationship between TMAO and skeletal muscle therefore requires consideration of microbial metabolism, host biotransformation, and target tissue exposure (29, 30). In a human isotope-tracing study, an isotope signal was detected in skeletal muscle following ingestion of labeled TMAO, supporting its delivery to muscle tissue (31). However, this exposure evidence alone does not establish a functional effect on muscle.

Human observational studies have mainly examined TMAO in relation to frailty and physical function in older adults. A cross-sectional study of 451 older patients with cardiovascular disease reported higher circulating TMAO levels in those with frailty, and this association persisted after adjustment for age, sex, and selected disease-related confounders (32). However, the main frailty outcome was assessed using a 48-item deficit-accumulation frailty index rather than an isolated measure of muscle mass or strength. The findings therefore support an association with frailty without establishing a muscle-specific relationship or its direction of causality (32).

Animal studies suggest that TMAO may modify susceptibility to inflammatory stress. In mice receiving intraperitoneal TMAO administration for four weeks, TMAO reduced intestinal claudin-1 staining intensity, suggesting altered expression or distribution of this tight junction protein. This finding alone does not establish increased intestinal permeability. TMAO alone did not significantly alter the frailty index, but mice pretreated with TMAO developed a more pronounced and prolonged frailty response following a subsequent LPS challenge (33). These findings associate TMAO exposure with changes in an intestinal barrier–related marker and greater vulnerability to inflammatory stress, although they do not establish that the barrier changes mediated the functional decline (33).

Experimental findings on the effects of TMAO in muscle are heterogeneous. In C2C12 cells subjected to oxidative stress, TMAO activated nuclear factor erythroid 2-related factor 2 (Nrf2) signaling, increased the expression of downstream antioxidant enzymes, including heme oxygenase-1 (HO-1) and NAD(P)H quinone dehydrogenase 1 (NQO1), and reduced cellular injury. Nrf2 inhibition abolished this protection. The corresponding animal experiments also showed enhanced antioxidant responses in skeletal muscle and improved swimming endurance (34). These results indicate that TMAO can exert cytoprotective effects under specific experimental conditions, arguing against a uniformly proinflammatory or proatrophic interpretation of its muscle actions (34). Conversely, in aged rats fed a high-fat diet, TMAO supplementation aggravated sarcopenic obesity and was accompanied by greater skeletal muscle mitochondrial dysfunction, increased reactive oxygen species production, and reduced Akt/mTOR signaling. These findings suggest adverse muscle effects under conditions of aging and metabolic stress, although they do not establish the same effects in primary age-related sarcopenia in humans (35).

The available literature provides evidence of TMAO exposure in skeletal muscle, observational associations with frailty, and functional effects in cell and animal models. However, study populations, exposure regimens, and outcomes differ substantially, and these findings do not yet constitute a continuous causal chain from microbial metabolism to muscle dysfunction. TMAO should therefore be regarded as a context-dependent candidate mediator rather than an established causal factor in sarcopenia. Effects involving NF-κB, the NLR family pyrin domain containing 3 (NLRP3) inflammasome, or fibrosis reported in other organs require muscle-specific validation before they can be incorporated into mechanistic accounts of sarcopenia. Further studies should connect microbial TMA production capacity and circulating TMAO levels with muscle metabolism and strength, while testing whether targeted modulation of this co-metabolic pathway alters muscle phenotypes.

## 3 Gut microbial alterations associated with sarcopenia

In recent years, the regulation of sarcopenia onset and progression by the gut microbiota via the gut-muscle axis has emerged as a prominent research hotspot in the fields of geriatrics and muscle physiology. Existing studies have largely been limited to descriptive and fragmented reporting of individual microbial features, lacking systematic integration of the intrinsic mechanistic framework underlying microbiota dysbiosis. Human studies have reported differences in gut microbial composition and function in sarcopenia, but findings vary with diagnostic definitions, comorbidities, medication exposure, diet, sampling, and analytical methods. Table 1 summarizes representative reports. The following synthesis examines their consistency and limitations and relates them to the experimental pathways described in Section 2.

### 3.1 Conceptual framework of gut microbial dysregulation

We propose a working framework linking microbial ecological changes, altered metabolic support, inflammatory responses, and muscle homeostasis. Changes in diet and nutritional status may accompany shifts in microbial composition (7, 47). Lower abundance of selected SCFA-associated taxa could reduce metabolic support if accompanied by reduced metabolite availability, while impaired colonization resistance could facilitate pathobiont expansion and barrier dysfunction (65–67). These processes may interact rather than follow a fixed temporal sequence. Human observational evidence and experimental findings support investigation of these links, but do not establish a unified causal pathway in human sarcopenia (12, 13, 25, 26, 68).

The framework distinguishes taxonomic composition, functional potential, and measured metabolic activity. Functional redundancy describes the capacity of different community members to contribute similar functions (69); neither its loss nor impaired ecological resilience can be inferred from taxonomic differences alone. Comparable α-diversity can coexist with differences in species composition and annotated pathways (46). Likewise, reduced abundance of SCFA-associated taxa does not necessarily indicate reduced SCFA production, which depends on substrate availability and community interactions. Longitudinal and intervention studies should test whether these features precede, accompany, or follow muscle decline.

### 3.2 Synthesis of human microbial findings

#### 3.2.1 Microbial diversity and population heterogeneity

Findings for gut microbial α-diversity in sarcopenia are inconsistent, and compositional differences can occur without a detectable reduction in richness or diversity. Diversity indices should therefore be interpreted alongside species-level profiles, annotated microbial functions, and measured metabolites. The available cross-sectional evidence does not establish which of these measures, if any, predicts subsequent muscle decline (46, 68).

In the population-based Xiangya Sarcopenia Study, shotgun metagenomics identified differences in microbial community composition between participants with and without sarcopenia, while Shannon α-diversity was not associated with sarcopenia (46). Differences in selected functional pathways were also reported, although several comparisons did not meet a false-discovery-adjusted threshold of 0.05 and remain exploratory. These findings illustrate why similar α-diversity does not imply equivalent microbial composition or function; they do not establish loss of functional redundancy or the value of a microbial profile as an early diagnostic marker.

Population characteristics may contribute to heterogeneous findings. In a Japanese outpatient study, men with sarcopenia had lower α-diversity and distinct β-diversity profiles, whereas corresponding differences were not observed in women (51). In older Uyghur adults, sarcopenia was associated with differences in microbial composition despite no significant differences in the assessed α-diversity indices (45). A separate study of healthy older adults found lower bacterial Simpson diversity in those aged 75–89 years than in those aged 60–74 or 90–99 years, with no significant difference between the latter two groups (70). These age-related findings are not specific to sarcopenia. Comorbidities, medications, and diet may also influence microbial composition (5–7), but their independent contributions to diversity differences in sarcopenia remain uncertain. Such factors should be measured and addressed analytically rather than assumed to explain between-study differences.

#### 3.2.2 Taxon-specific alterations in SCFA-associated bacteria

Reported associations between gut microbial taxa and sarcopenia vary across studies, including inconsistent findings for genera such as *Lactobacillus* and *Bifidobacterium* (68). These findings suggest that sarcopenia-associated microbial alterations should be examined at the level of individual taxa and their metabolic functions, rather than interpreted as a uniform reduction in all taxa commonly regarded as beneficial. Differences in the taxa assessed and the populations investigated may contribute to between-study heterogeneity (68).

Selected SCFA-associated taxa have been reported at lower relative abundances in some studies of sarcopenia or low muscle mass. A small exploratory shotgun metagenomic study reported lower relative abundances of selected SCFA-associated species and reduced representation of genes involved in SCFA synthesis (39). These findings reflect functional potential rather than gene expression or metabolic flux. Separately, lower fecal butyrate concentrations were reported in older adults with low skeletal muscle mass (61). These observations motivate investigation of SCFA metabolism but do not establish a uniform metabolic defect across sarcopenia populations.

*Faecalibacterium prausnitzii* is an important butyrate-producing commensal in the human intestinal tract (8, 9). A small exploratory metagenomic study reported a lower relative abundance of this species in participants with sarcopenia, although its consistency across populations requires further validation (39). Experimental evidence further suggests a potential role in maintaining intestinal barrier integrity and muscle mitochondrial function: supplementation with *F. prausnitzii* improved colonic tight junction protein expression, muscle mitochondrial parameters, and muscle mass and function in aged mice (71). These findings support its potential relevance to the gut–muscle axis, while its causal role in human sarcopenia remains to be established.

The metabolic contributions of propionate-associated taxa require species- and substrate-specific interpretation. Certain Lachnospiraceae species, including *Roseburia inulinivorans*, can produce propionate from particular substrates (72). *Prevotella* should not be uniformly classified as propionate-producing: *P. copri* has been reported to produce succinate, which can serve as a substrate for propionate production by other community members (73). Family- or genus-level abundance therefore cannot be treated as a direct measure of propionate production. In an acute intervention study in overweight adults, targeted colonic propionate delivery increased peptide YY (PYY) and glucagon-like peptide-1 (GLP-1) release and reduced energy intake (74). This finding does not establish that depletion of propionate-associated bacteria reduces protein intake in older adults with sarcopenia.

Together, these studies identify SCFA-associated taxa as candidates for further investigation rather than validated microbial markers of sarcopenia. Taxonomic changes should be evaluated alongside functional and metabolite measurements before conclusions are drawn about reduced SCFA-mediated support.

#### 3.2.3 Pathobionts and metabolites in the interpretation of human findings

An increased relative abundance of a putative pathobiont does not, by itself, demonstrate greater endotoxin exposure or muscle injury. Microbiota-mediated colonization resistance provides a biological rationale for examining such associations (67), but strain identity, intestinal barrier function, and circulating microbial products must be measured to connect taxonomic observations to the inflammatory mechanisms described in Section 2.5.

For example, strains of *Escherichia coli* and *Klebsiella pneumoniae* have been identified as intestinal endotoxin producers in experimental fatty liver disease (75), and preparations derived from *Parabacteroides distasonis* impaired epithelial barrier function in work on early-life antibiotic exposure and hepatic steatosis (76). These findings cannot be generalized to entire genera or directly extrapolated to human sarcopenia. Downstream experiments show that LPS can induce catabolic signaling in muscle cells and mice (25, 26) and transiently inhibit protein synthesis in C2C12 myotubes (27). The unresolved link is whether the microbial differences observed in human sarcopenia generate physiologically relevant exposures sufficient to produce these responses.

Taxonomic findings also require functional validation for the TMA/TMAO pathway. Some members of the Enterobacteriaceae harbor genes encoding choline TMA-lyase (CutC), but their relative abundance alone does not quantify TMA production or host TMAO exposure (29, 77). As discussed in Section 2.6, experimental muscle responses to TMAO vary with host and exposure conditions (34, 35), and observational associations with frailty do not establish causation in sarcopenia (32). Studies should therefore connect microbial metabolic capacity with measured circulating metabolites and muscle outcomes rather than infer harmful effects from bacterial abundance alone.

#### 3.2.4 Microbial functional potential and broad taxonomic indicators

Metagenomic studies have identified differences in microbial functional potential in sarcopenia, including altered representation of genes involved in SCFA synthesis and amino acid biosynthesis (39, 46). However, these findings do not establish global suppression of anabolic pathways or universal activation of catabolic and pro-inflammatory pathways. Moreover, microbial gene abundance does not directly measure gene expression, metabolic flux, or host skeletal muscle signaling.

The two metagenomic studies illustrate why pathway-level findings should be described separately rather than summarized as a single loss of microbial function. In a proof-of-concept study of 17 older outpatients (five with sarcopenia and 12 controls), the sarcopenia group had lower representation of pathways including pyruvate fermentation to acetate, succinate fermentation to butanoate, and biosynthesis of methionine, lysine, and threonine. Genes associated with glycolysis and the glyoxylate bypass were more represented, indicating that the observed differences were not uniformly downward (39). The small, cross-sectional sample limits both generalizability and causal interpretation.

In the community-based Xiangya Sarcopenia Study, shotgun metagenomic profiling of 1,417 participants showed lower representation of phenylalanine, tyrosine, and tryptophan biosynthesis in those with sarcopenia, alongside higher representation of selected pathways, including alpha-linolenic acid metabolism and furfural degradation (46). Several of these pathway comparisons had false-discovery-adjusted *Q* values above 0.05 and should be regarded as exploratory. The direction of the reported associations varied by pathway and cohort. As these were inferred from fecal microbial DNA, neither study demonstrated corresponding changes in microbial transcription, circulating amino acid availability, or skeletal muscle protein turnover (39, 46). A separate study found lower measured fecal butyrate in older adults with low skeletal muscle mass (61), but that metabolite finding cannot be treated as a direct validation of pathway estimates in the other cohorts.

The Firmicutes/Bacteroidetes (F/B) ratio combines organisms with different metabolic capacities into two phylum-level totals. Communities with similar ratios can differ in SCFA-associated species and relevant pathways; conversely, a change in the ratio does not identify the species or processes responsible. Species- and pathway-level findings therefore provide information that this broad indicator cannot resolve (39, 46).

Underlying diseases, medication use, and diet should be considered when comparing F/B ratios across clinical and community-based populations (5–7). However, the available evidence does not establish that hospitalization or antibiotic exposure explains the direction of reported differences in sarcopenia. The ratio has not been validated as a standalone clinical biomarker.

A more informative assessment would report species-level profiles and annotated pathways alongside measured fecal or circulating metabolites and standardized muscle outcomes. Dietary intake, medication use, and comorbid disease should be documented because each can influence the microbial signal (5–7). Longitudinal sampling and validation in independent populations are needed before any functional profile, metabolite panel, or F/B ratio can be used to predict sarcopenia or to infer a causal gut–muscle pathway.

## 4 Interventions targeting the gut microbiota and its metabolites in sarcopenia

### 4.1 Probiotics and prebiotics

Effects of probiotic supplementation on muscle health depend on the strain, population, and outcome assessed. A randomized, double-blind trial of *Lactobacillus plantarum* TWK10 in frail older adults reported improvements in selected muscle-related outcomes, although some findings reflected within-group changes rather than significant differences from placebo (78). This trial provides preliminary human evidence, but frailty is not interchangeable with a confirmed diagnosis of sarcopenia. Larger trials in well-defined sarcopenia populations are needed to establish clinical benefit.

Other cited bacterial interventions were evaluated in preclinical models. *Bifidobacterium longum* OLP-01 and *Lactobacillus salivarius* SA-03 improved selected measures of exercise performance or fatigue in mice (79, 80), while *Lactobacillus casei* Shirota attenuated age-related muscle deficits in SAMP8 mice (81). *Faecalibacterium prausnitzii* alleviated muscle atrophy in a rat model of chronic renal failure with protein-energy wasting, with experimental evidence implicating mitophagy regulation (82). *Bifidobacterium adolescentis* and its metabolite nicotinic acid improved mitochondrial function and muscle outcomes in aged mice (19). These results support strain-specific investigation but do not establish efficacy in older adults with sarcopenia or justify treating all tested bacteria as clinically validated probiotics.

Prebiotics are substrates selectively utilized by host microorganisms that confer a health benefit; examples include inulin and fructooligosaccharides (83). Their effects depend on substrate characteristics and the metabolic capabilities of the resident microbiota (10). Inulin supplementation was associated with increased fat-free mass and changes in muscle-related biomarkers in children with obesity (84), and alleviated skeletal muscle atrophy in burn-injured rats (85). Neither population directly represents older adults with sarcopenia. The evidence summarized here does not establish an optimal strain or substrate, dose, treatment duration, or long-term clinical benefit. Adequately powered randomized controlled trials should assess muscle strength and physical performance as well as body composition.

### 4.2 Fecal microbiota transplantation

Fecal microbiota transplantation (FMT) transfers fecal microbial communities from screened donors to recipients with the aim of modifying the recipient microbiota (86). Experimental studies of young-donor microbiota transplantation in aged mice have reported improvements in selected measures of frailty, physical fitness, or metabolic health (87–89). Outcomes and microbial or metabolic changes differ across models, and these findings do not establish a single shared mechanism or consistent benefit across all muscle outcomes.

A retrospective study compared 85 patients receiving FMT plus resistance training with 87 receiving resistance training alone over 24 weeks and reported improvements in muscle-related and inflammatory outcomes with the combined intervention (90). The nonrandomized design limits causal interpretation and does not establish that changes in SCFAs or other microbial metabolites mediated the outcomes. Clinical evidence remains preliminary. Sarcopenia-specific trials are needed to define patient selection, donor screening, administration protocols, durability of benefit, and short- and long-term safety before FMT can be considered for routine management.

### 4.3 Microbiota-guided interventions for future testing

High-throughput sequencing can characterize gut microbial composition, while shotgun metagenomics can assess functional potential; complementary metabolomic measurements are needed to identify metabolite abnormalities associated with sarcopenia (42, 46). These findings may inform the design of personalized interventions for future testing. In individuals with reduced abundance of selected SCFA-associated taxa and independently confirmed low SCFA availability, targeted probiotic or prebiotic supplementation could be investigated. For individuals with elevated TMAO levels, approaches targeting microbial metabolism of dietary precursors, including selected probiotic interventions, could be investigated, but their efficacy in sarcopenia remains unestablished (29, 30, 91). Dietary fiber intake is another potential intervention target, although its effects on microbial composition and muscle outcomes in older adults with sarcopenia require direct evaluation (7, 83, 85). Longitudinal monitoring of gut microbiota and metabolites may help assess biological responses in intervention studies, but its value for guiding routine sarcopenia management remains to be established.

## 5 Research outlook

Three challenges limit clinical translation of gut–muscle research. First, most human studies are cross-sectional and cannot determine whether microbial alterations precede or follow muscle decline (68). Second, microbial signatures vary across populations and methods, and reproducible biomarkers have not been validated for clinical use. Third, intervention studies have not established effective regimens, suitable target populations, or long-term safety. This narrative review also has limitations: evidence selection was not exhaustive, no formal risk-of-bias assessment was performed, and possible overlap between human research reports was not resolved. The synthesis should therefore be interpreted as a qualitative appraisal of candidate mechanisms rather than a quantitative estimate of the total evidence.

Future research should prioritize longitudinal cohorts that integrate metagenomics, metabolomics, dietary assessment, medication exposure, and repeated measurements of muscle outcomes. Mechanistic studies using germ-free or defined-community models can test candidate pathways identified in humans, while genetic and causal-inference approaches may provide complementary evidence when their assumptions are met. Finally, adequately powered randomized controlled trials should evaluate strain- or metabolite-specific interventions using standardized sarcopenia definitions and clinically meaningful outcomes, including muscle strength and physical performance.

## 6 Conclusion

Gut microbial functions and microbiota-related metabolites are plausible contributors to muscle homeostasis, with experimental support for pathways involving SCFAs, nicotinic acid, amino acid metabolism, bile acid signaling, and inflammatory responses (4, 13, 19, 20). Human associations remain heterogeneous, and the causal contribution of these pathways to sarcopenia is not established. Probiotics, prebiotics, FMT, and microbiota-guided approaches remain investigational for this indication. Progress requires longitudinal studies and targeted trials that connect microbial functions and measured metabolites with muscle strength, physical performance, and other clinically meaningful outcomes.
